# LncRNA DANCR attenuates brain microvascular endothelial cell damage induced by oxygen-glucose deprivation through regulating of miR-33a-5p/XBP1s

**DOI:** 10.18632/aging.102712

**Published:** 2020-01-26

**Authors:** Mengqi Zhang, Mimi Tang, Qian Wu, Zhuolu Wang, Zhuohui Chen, Hui Ding, Xinhang Hu, Xinyi Lv, Songfeng Zhao, Jingyan Sun, Shuntong Kang, Tong Wu, Bo Xiao

**Affiliations:** 1 Department of Neurology, Xiangya Hospital, Central South University, Changsha 410008, China; 2 Department of Pharmacy, Xiangya Hospital, Central South University, Changsha 410008, China; 3 Department of Neurology, First Affiliated Hospital, Kunming Medical University, Kunming 650032, China; 4 Department of Breast Surgery, Hunan Provincial Maternal and Child Health Care Hospital, Changsha 410008, China

**Keywords:** DANCR, miR-33a-5p, X-box binding protein l splicing, ischemic stroke, brain microvascular endothelial cell

## Abstract

Brain microvascular endothelial cell (BMEC) survival and angiogenesis after ischemic stroke has great significance for improving the prognosis of stroke. Abnormal variants of lncRNAs are closely associated with stroke. In this study, we examined the effects and molecular mechanisms of differentiation antagonizing non-protein coding RNA (DANCR) on apoptosis, migration, and angiogenesis of oxygen-glucose deprivation (OGD)-treated BMECs. We found that DANCR expression significantly increased at 2, 4, 6, 8, and 10 h after OGD. DANCR overexpression promoted cell viability, migration, and angiogenesis in OGD-treated BMECs. Additionally, we found that X-box binding protein l splicing (XBP1s) expression was positively correlated with DANCR expression. DANCR overexpression promoted XBP1s expression in OGD-treated BMECs. Silenced XBP1s reversed the effect of DANCR in OGD-treated BMECs. Furthermore, we found that microRNA (miR)-33a-5p bound to DANCR and the 3'-UTR of XBP1. miR-33a-5p overexpression inhibited proliferation, migration, angiogenesis, and XBP1s expression in OGD-treated DANCR-overexpressing BMECs, reversing the protective effect of DANCR. Finally, we found that XBP1s expression promoted proliferation, migration, and angiogenesis, reversing the damaging effect of miR-33a-5p. In conclusion, DANCR enhanced survival and angiogenesis in OGD-treated BMECs through the miR-33a-5p/XBP1s axis.

## INTRODUCTION

Stroke causes neurological dysfunction and long-term disability, has a high incidence, and imposes an extremely heavy burden worldwide including in China [1, 2]. The two main types of stroke are hemorrhagic and ischemic stroke [3]. Ischemic stroke accounts for approximately 85% of all stroke cases. Although much effort is being put into the development of new treatment strategies for ischemic stroke, thrombolytic therapy is currently the only effective treatment [4]. Identification of novel and effective therapies is, therefore, of paramount importance, and further understanding of the pathological course will greatly assist these efforts.

Non-coding RNAs (ncRNAs), including microRNAs (miRNAs) and long ncRNAs (lncRNAs), modulate gene expression and function through different mechanisms [5, 6]. lncRNAs may act as competing endogenous RNAs (ceRNAs) by interacting with miRNAs and regulating the expression of the miRNA target protein. Abnormal variants of miRNAs and lncRNAs are closely associated with stroke [7, 8]. ANRIL reduced apoptosis from cerebral ischemia-induced injury in PC-12 cells through the miR-127/Mcl-1 axis [9]. Additionally, in cerebral ischemic stroke, KCNQ1OT1 knockdown significantly decreases the infarct volume and improves neurological function by regulating miR-200a/FOXO3 and promoting autophagy [10]. Briefly, lncRNAs play a critical role in protecting cerebral microvasculature against cerebral ischemic insults. Differentiation antagonizing non-protein coding RNA (DANCR) is an lncRNA located on chromosome 4q12 that maintains stemness and suppresses progenitor differentiation [11]. DANCR promotes tumor progression and angiogenesis in glioma [12] and stimulates glioma proliferation by activating the WNT/β-catenin pathway [13]. However, the role played by DANCR in ischemic stroke remains unclear.

Various studies have shown that neuro-protectants are effective in animal models of stroke, although they have shown less effectiveness in the clinical research of stroke, suggesting that focusing only on neuro-protection is inadequate [14, 15]. Hence, microenvironment of neurons, including brain microvasculature, vascular neural network, and neurovascular units, has recently received much attention [16]. Brain microvascular endothelial cells (BMECs) are vital components of the cerebral microvasculature; they form the blood–brain barrier (BBB) and play a dominant role in maintaining its integrity and in brain homeostasis [17, 18]. Ischemic stroke leads to BMEC death, which induces BBB disruption and enhances vascular permeability, resulting in brain edema formation and development and a poor prognosis for patients with ischemic stroke [19, 20]. Therefore, protecting the BMECs against ischemia-induced injury can improve the prognosis of patients with ischemic stroke. Hence, we studied the effects of DANCR on the apoptosis, migration, and angiogenesis of OGD-treated BMECs. In addition, we investigated molecular mechanisms underlying the effects of DANCR on cell viability, cell migration, and angiogenesis of OGD-treated BMECs.

## RESULTS

### Altered DANCR expression in OGD-treated BMECs

**Figure 1:**
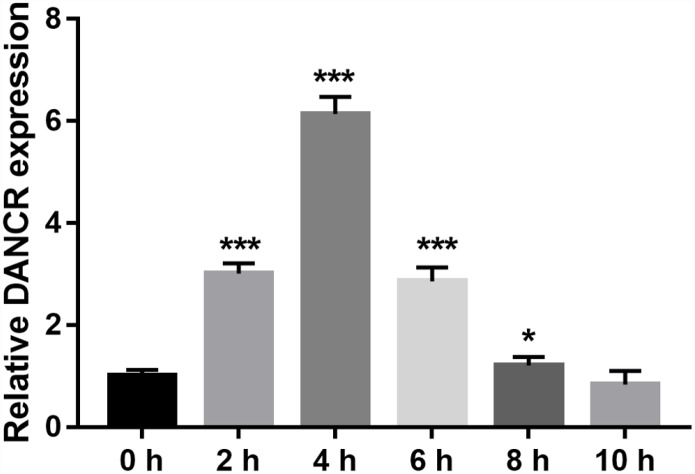
**DANCR expression increased after OGD treatment.** DANCR expression was measured using qRT-PCR after OGD treatment for 0, 2, 4, 6, 8, and 10 h. *P<0.05, ***P<0.001.

DANCR expression was measured using qRT-PCR. DANCR expression levels were the highest at 4 h and the lowest at 10 h after OGD treatment (Figure 1). Additionally, DANCR expression significantly increased at 2, 4, 6, and 8 h after OGD treatment (Figure 1). We chose to treat the BMECs with OGD for 4 h to establish an OGD-treated BMEC model.

### DANCR overexpression promoted proliferation, migration, and angiogenesis of OGD-treated BMECs

**Figure 2:**
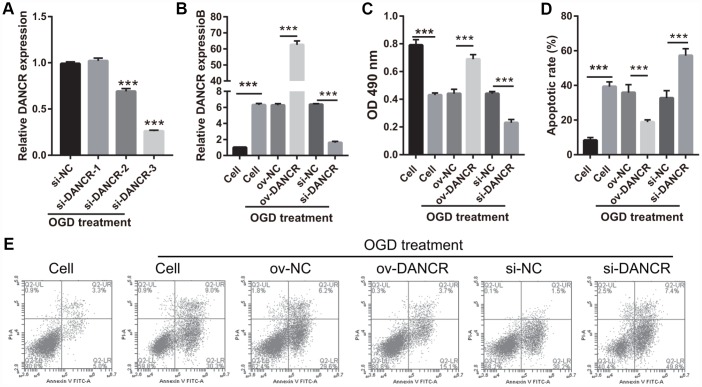
**DANCR overexpression promoted proliferation in OGD-treated BMECs.** (**A**) DANCR expression was measured using qRT-PCR after transfection with si-DANCR-1/2/3 at 48 h and then treated with OGD for 4 h. (**B**) DANCR expression was measured using qRT-PCR after transfection with DANCR-pcDNA3.1 or si-DANCR at 48 h and then treated with OGD for 4 h. (**C**) Proliferation was measured by MTS after transfection at 48 h, followed by OGD treatment for 4 h. (**D**) The bar represents the apoptotic rate. (**E**) Representative image of apoptosis as measured by flow cytometry after transfection at 48 h, followed by OGD treatment for 4 h. ***P<0.001.

**Figure 3:**
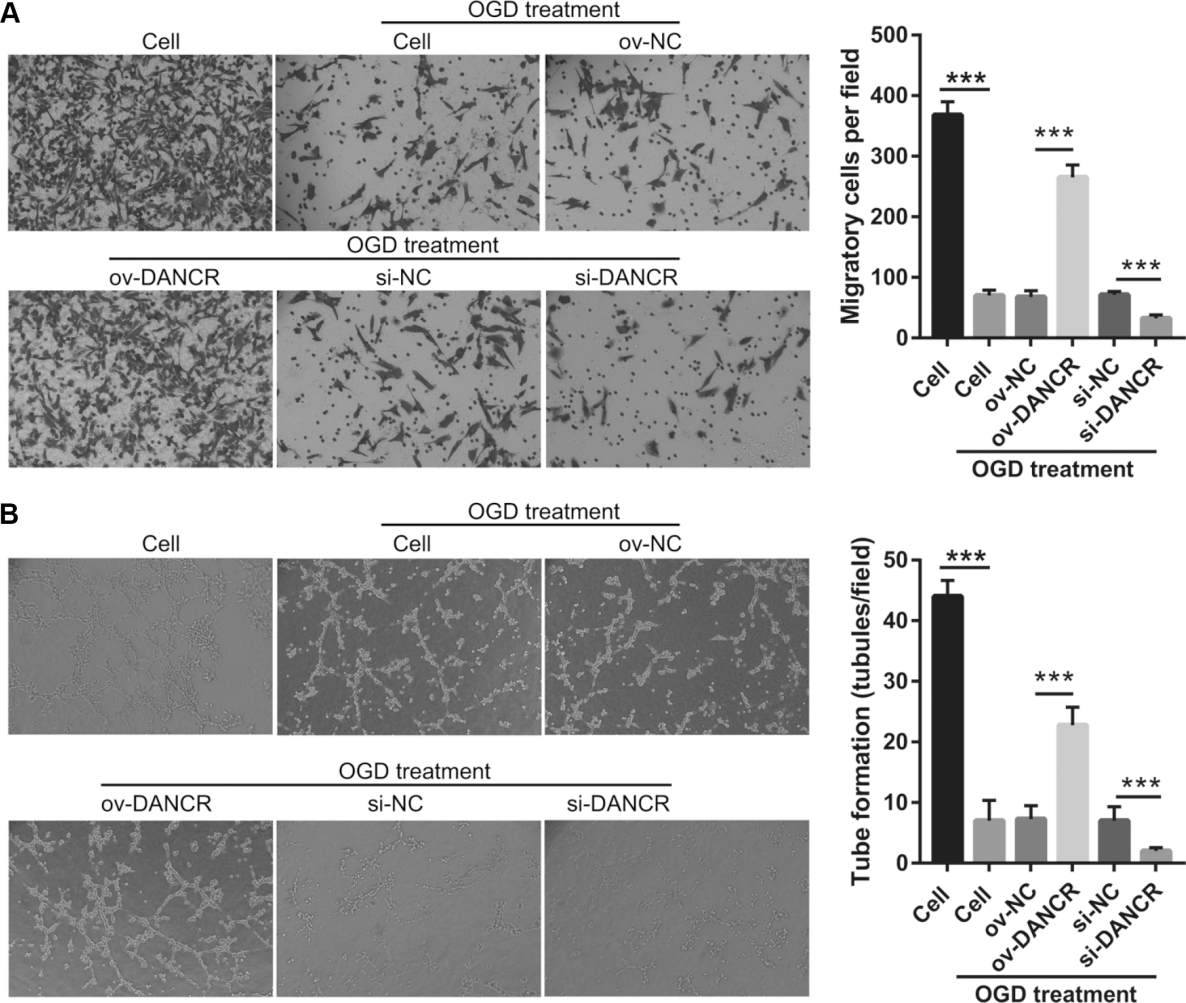
**DANCR overexpression promotes migration and angiogenesis in OGD-treated BMECs.** (**A**) Representative image of the migration (scale bar: 200×) measured transwell after transfection at 48 h followed by OGD treatment for 4 h. The bar represents migratory cells. (**B**) Representative image of angiogenesis (scale bar: 200×) measured by tube formation assay after transfection at 48 h followed by OGD treatment for 4 h. The bar represents the number of meshes. ***P<0.001.

After the optimization of the OGD-treated BMEC model, we explored the effect of DANCR on cell proliferation. BMECs were first transfected with DANCR-pcDNA3.1 and si-DANCR at 48 h, and we evaluated DANCR mRNA expression using qRT-PCR after the 4-h OGD treatment. DANCR expression was significantly inhibited in cells transfected with si-DANCR-2 and, particularly, si-DANCR-3, compared with si-NC-transfected cells (Figure 2A). Thus, si-DANCR-3 was used as the siRNA (si-DANCR) in subsequent experiments. From the results (Figure 2B), it can be concluded that BMECs were successfully transfected with DANCR and at a higher rate than that of the OGD-treated BMECs transfected with the negative control (ov-NC). Next, MTS assay showed that cell viability of the DANCR group was significantly increased compared with that of the ov-NC groups, whereas that of the si-DANCR group was significantly decreased compared with that of the si-NC group (Figure 2C). It can be concluded from our results that DANCR overexpression can effectively inhibit apoptosis of OGD-treated BMECs, while silenced DANCR can effectively enhance apoptosis (Figures 2D and 2E). To further evaluate the effect of DANCR on BMECs, cell migration and tube formation assays were performed. From Figure 3A, it can be observed that the migration ability of cells in the DANCR group was much higher than that of cells in the ov-NC group, while silenced DANCR significantly decreased the migration ability. Moreover, angiogenesis was also promoted in the DANCR group and inhibited in the si-DANCR group, as shown in Figure 3B.

### DANCR regulates the proliferation and angiogenesis of OGD-treated BMECs by X-box binding protein l splicing (XBP1s)

**Figure 4:**
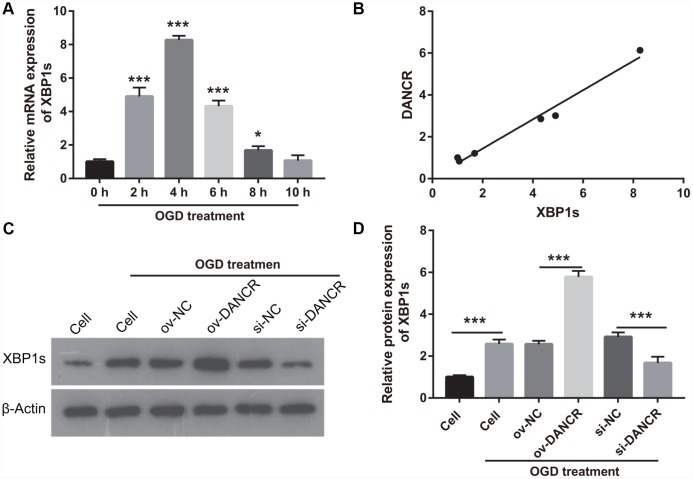
**DANCR enhanced XBP1s expression in OGD treated BMECs.** (**A**) XBP1s expression was measured using qRT-PCR after OGD treatment for 0, 2, 4, 6, 8, and 10 h. (**B**) DANCR expression was positively correlated with XBP1s expression. The relationship between DANCR expression and XBP1s expression was analyzed using Pearson’s analysis. (**C**) XBP1s expression were measured using western blot after transfection with DANCR-pcDNA3.1 and si-DANCR at 48 h and then treated with OGD for 4 h. (**D**) The bar represents the protein expression of XBP1s. *P<0.05, ***P<0.001.

**Figure 5:**
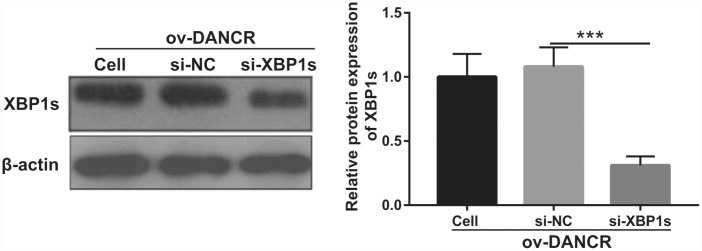
**XBP1s expression was inhibited by transfection with si-XBP1.** XBP1s expression was measured using western blot after co-transfection with DANCR-pcDNA3.1 and si-XBP1 at 48 h and then treated with OGD for 4 h. ***P<0.001.

**Figure 6:**
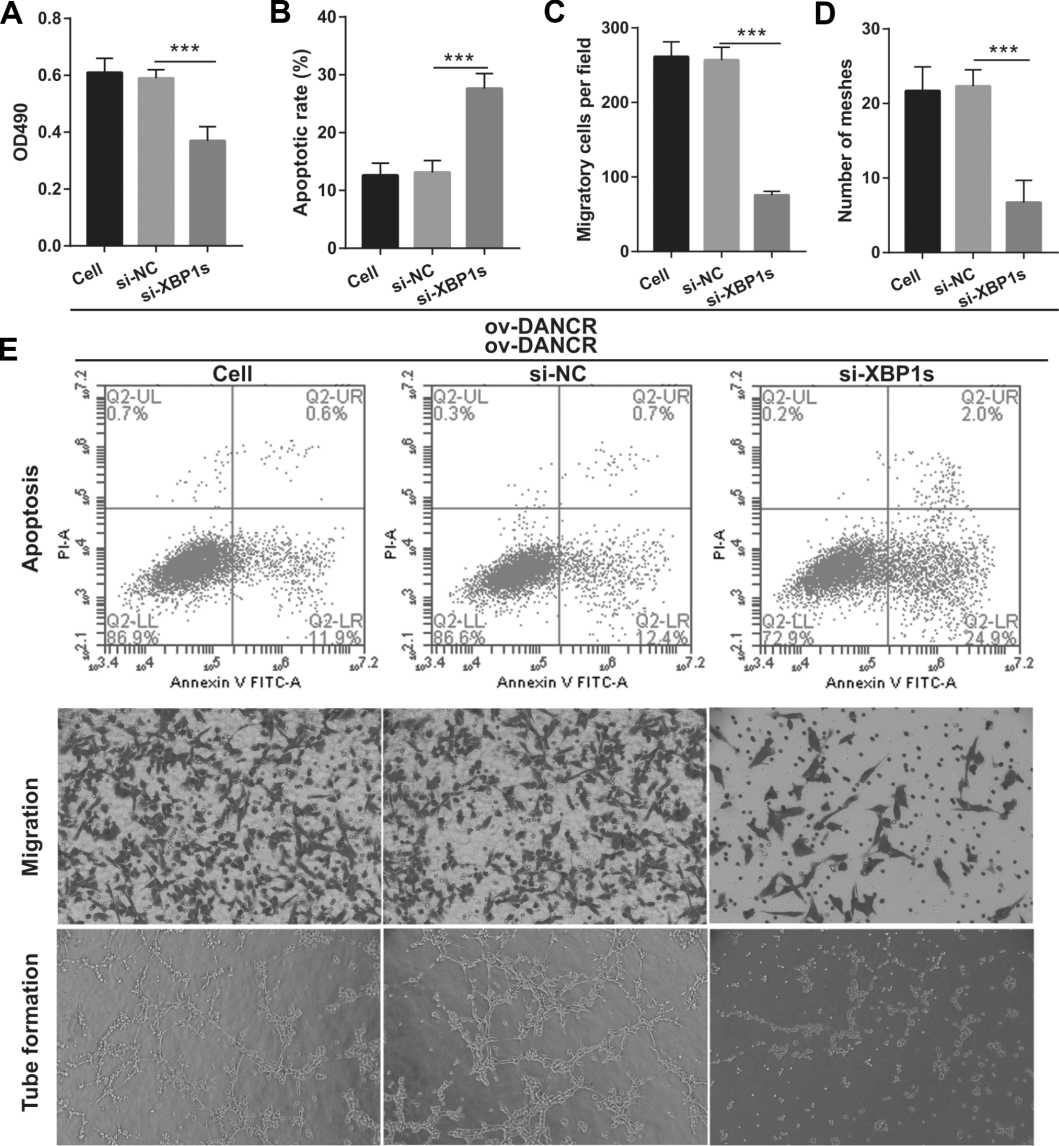
**Silencing of XBP1s reversed the effect of DANCR on proliferation and angiogenesis of OGD-treated BMECs.** (**A**) Proliferation was measured by MTS after co-transfection with DANCR-pcDNA3.1 and si-XBP1 at 48 h, followed by OGD treatment for 4 h. (**B**–**D**) The bar represents the apoptotic rate (**B**), migratory cells (**C**), and number of meshes (**D**). (**E**) A representative image of apoptosis, migration (scale bar: 200×), and angiogenesis (scale bar: 200×), measured by flow cytometry, transwell, and tube formation assay, respectively, after co-transfection with DANCR-pcDNA3.1 and si-XBP1 at 48 h, followed by OGD treatment for 4 h. ***P<0.001 vs ov-NC.

In our previous study, we found that XBP1s promotes BMEC survival and induces angiogenesis, thereby attenuating ischemia-induced BMEC injury [21]. In the present study, we examined the effect of DANCR on XBP1s expression. First, XBP1s expression was significantly increased at 2, 4, 6, and 8 h after OGD treatment, which positively correlated with DANCR expression (Figure 4A and 4B). XBP1s expression was significantly promoted by DANCR overexpression, whereas it was significantly inhibited by silencing DANCR expression in OGD-treated BMECs (Figure 4C and 4D). We further evaluated the effect of Xbp1s on DANCR and found that XBP1s expression was significantly inhibited in the DANCR+si-XBP1 group (Figure 5). Proliferation, migration, and angiogenesis in cells of the DANCR+si-XBP1 group were much lowerthan those in cells of the DANCR and DANCR+si-NC groups, whereas apoptosis in the DANCR+si-XBP1 group was significantly promoted (Figure 6).

### DANCR and the 3′-UTR of XBP1 competitively combined with miR-33a-5p

**Figure 7:**
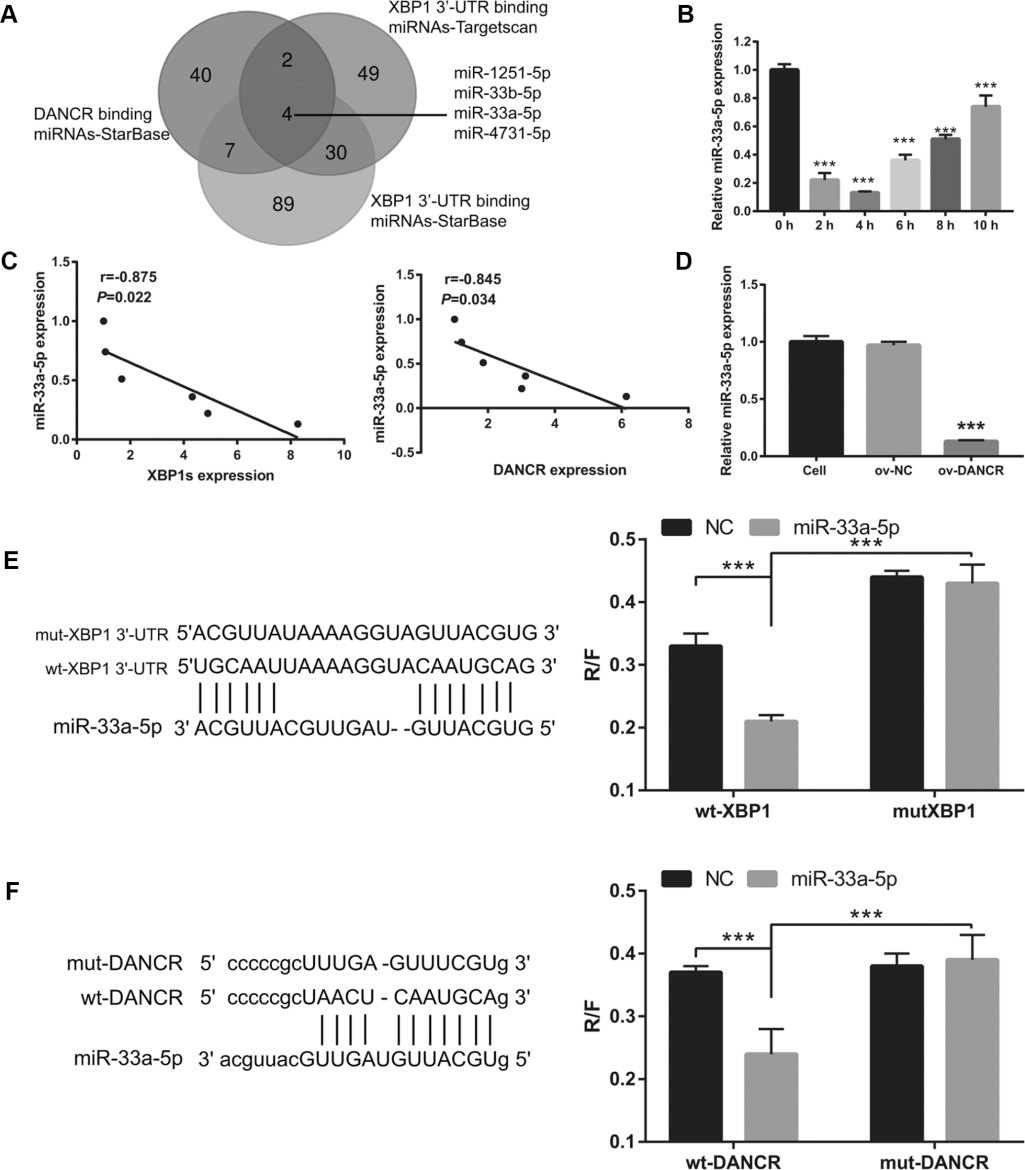
**miR-33a-5p directly bound to DNACR and XBP1.** (**A**) miRNAs bound to DNACR and XBP1 were analyzed using StarBase V2.0 and Targetscan V7.2. (**B**) miR-33a-5p expression was measured using qRT-PCR after OGD treatment for 0, 2, 4, 6, 8, and 10 h. (**C**) The relationship between miR-33a-5p expression and DANCR or XBP1s expression was analyzed using Pearson’s analysis. (**D**) miR-33a-5p expression was measured using qRT-PCR after transfection with DANCR-pcDNA3.1 at 48 h, followed by OGD treatment for 4 h. (**E**) Predicted binding sites between XBP1 and miR-33a-5p; luciferase reporter assay was conducted to detect luciferase activity after co-transfection of BMECs with WT-XBP1 or MUT-XBP1 and NC, miR-33a-5p mimics. (***P< 0.001). (**F**) Predicted binding sites between DANCR and miR-33a-5p; luciferase reporter assay was conducted to detect luciferase activity after co-transfection of BMECs with WT-DANCR or MUT-DANCR and NC, miR-33a-5p mimics. (***P< 0.001).

First, a bioinformatics prediction analysis was performed using StarBase V2.0 and Targetscan V7.2 to analyze miRNAs that can bind DANCR and XBP1. We found that DANCR and the 3′-UTR of XBP1 could competitively combine with miR-33a-5p, miR-33b-5p, miR-1251-5p, and miR-4731-5p (Figure 7A). Previous studies have found that DANCR can bind to miR-33a-5p [22, 23]. Therefore, we chose miR-33a-5p for further experiments. The results showed that miR-33a-5p expression was the lowest at 4 h and the highest at 10 h after OGD treatment (Figure 7B). Furthermore, miR-33a-5p expression negatively correlated with DANCR and XBP1s expression (Figure 7C). Additionally, DANCR overexpression significantly inhibited miR-33a-5p expression in OGD-treated BMECs (Figure 7D). Moreover, luciferase reporter assay showed that miR-33a-5p overexpression significantly decreased the WT-XBP1-associated luciferase activity compared to that in the NC group, whereas it had no obvious effect on the luciferase activity of MUT-XBP1 compared with that in the NC group (Figure 7E). These results also showed that miR-33a-5p overexpression significantly decreased the WT-DANCR-associated luciferase activity compared with that in the NC group, whereas it had no obvious effect on the luciferase activity of MUT-DANCR compared with that in the NC group (Figure 7F).

### miR-33a-5p overexpression reverses the effect of DANCR on the proliferation and angiogenesis of OGD-treated BMECs

**Figure 8:**
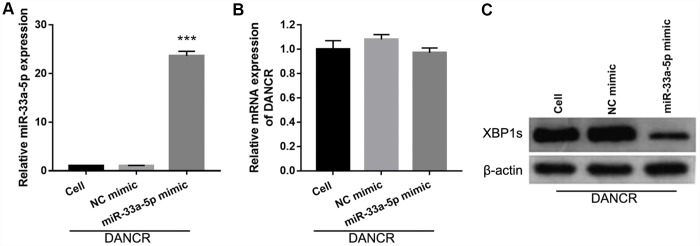
**miR-33a-5p mimic transfection promoted miR-33a-5p expression, inhibited XBP1s expression, and had no effect on DANCR expression in OGD-treated DANCR-overexpressing BMECs.** (**A**–**C**) miR-33a-5p expression (**A**) and DANCR expression (B) were measured using qRT-PCR, and XBP1s expression (**C**) was measured using western blot after co-transfection with DANCR-pcDNA3.1 and miR-33a-5p mimic at 48 h, followed by OGD treatment for 4 h.

**Figure 9:**
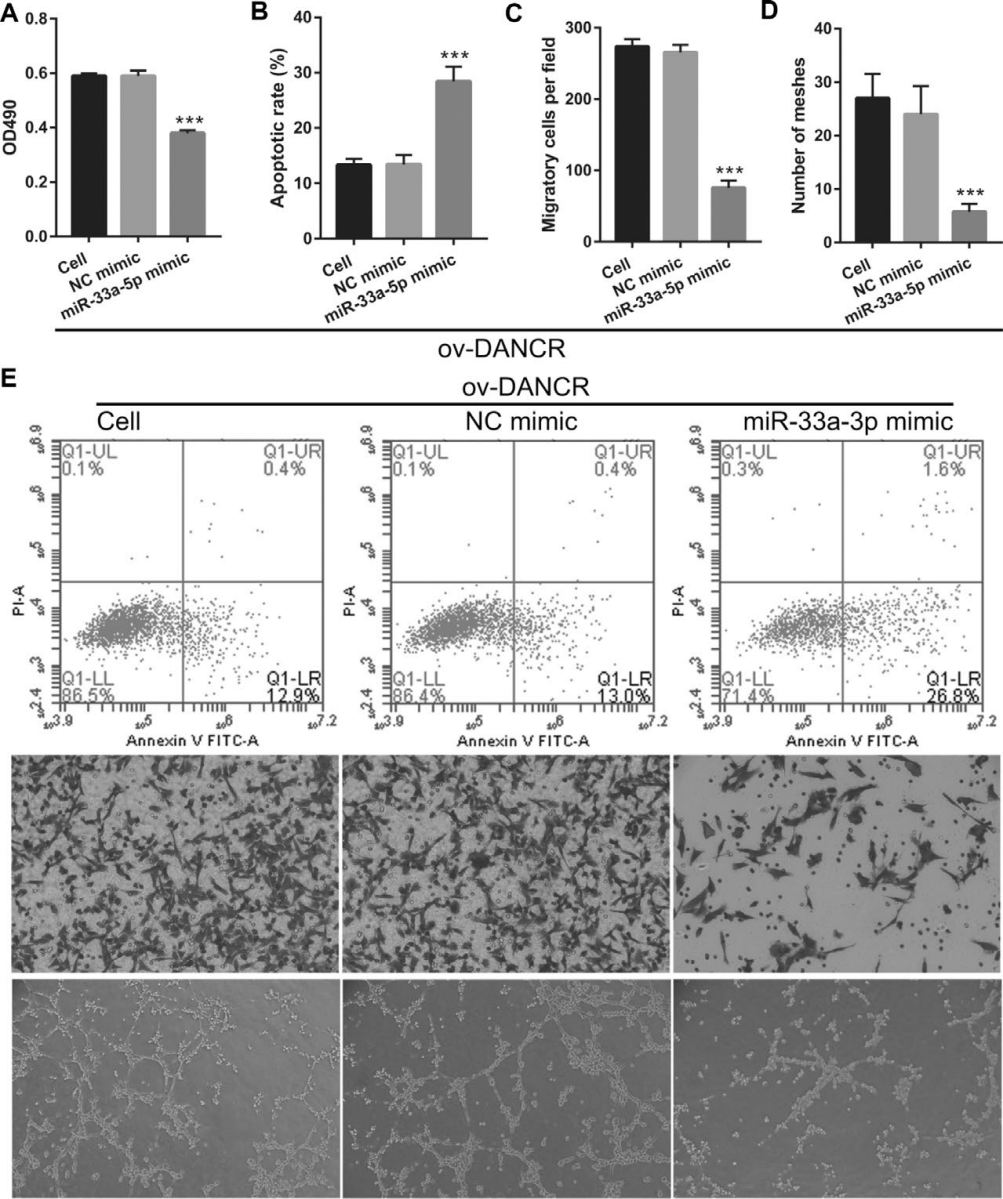
**miR-33a-5p overexpression reversed the effect of DANCR on proliferation and angiogenesis in OGD-treated BMECs.** (**A**) Proliferation was measured by MTS after co-transfection with DANCR-pcDNA3.1 and miR-33a-5p mimic at 48 h, followed by OGD treatment for 4 h. (**B**–**D**) The bar represents the apoptotic rate (**B**), migratory cells (**C**), and number of meshes (**D**). (**E**) A representative image of apoptosis, migration (scale bar: 200×), and angiogenesis (scale bar: 200×), measured by flow cytometry, trans-well, and tube formation assay, respectively, after co-transfection with DANCR-pcDNA3.1 and miR-33a-5p mimic at 48 h, followed by OGD treatment for 4 h. ***P<0.001 vs ov-NC.

To further study the influence of the interaction between miR-33a-5p and DANCR on OGD-treated BMECs, BMECs were transfected with both miR-33a-5p and DANCR. From the PCR results, we found that miR-33a-5p expression in the DANCR +miR-33a-5p mimic group was significantly higher than that in the DANCR+NC mimic group (Figure 8A). Compared to the DANCR+NC mimic group, DANCR expression was not significantly changed, whereas XBP1s expression was clearly reduced in the DANCR+miR-33a-5p mimic group (Figures 8B and 8C). Verification of our analysis revealed that cell viability, migration, and angiogenesis were significantly decreased, whereas apoptosis was significantly enhanced in the DANCR+miR-33a-5p group compared to the DANCR and DANCR+NC groups, owing to miR-33a-5p overexpression (Figure 9).

### XPB1s weakens the effect of miR-33a-5p on proliferation and angiogenesis in OGD-treated BMECs

**Figure 10:**
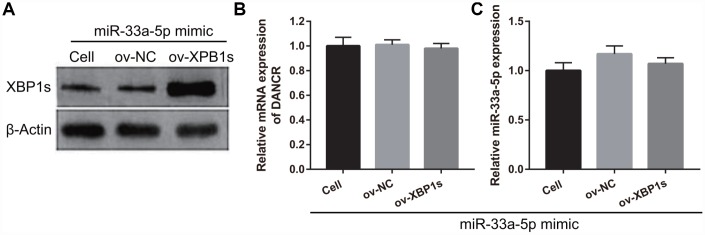
**XBP1s-pcDNA3.1 transfection promoted XBP1s expression and had no effect on DANCRor miR-33a-5p expression in miR-33a-5p-overexpressing BMECs after OGD-treatment.** (**A**–**C**) XBP1s expression (**A**) was measured using western blot and miR-33a-5p expression (**B**) and DANCR expression (**C**) were measured using qRT-PCR after co-transfection with XBP1s-pcDNA3.1 and miR-33a-5p mimic at 48 h, followed by OGD treatment for 4 h.

**Figure 11:**
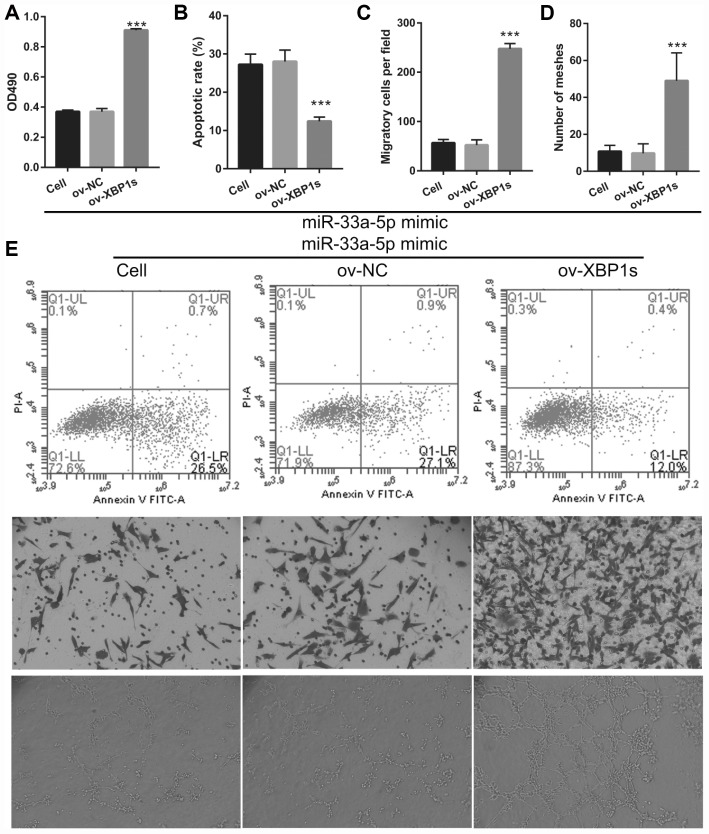
**XBP1s overexpression reversed the effect of miR-33a-5p on the proliferation and angiogenesis in OGD-treated BMECs.** (**A**) Proliferation was measured by MTS after co-transfection with miR-33a-5p mimic and XBP1s-pcDNA3.1 at 48 h, followed by OGD treatment for 4 h. (**B**–**D**) The bar represents the apoptotic rate (**B**), migratory cells (**C**), and number of meshes (**D**). (**E**) A representative image of apoptosis, migration (scale bar: 200×), and angiogenesis (scale bar: 200×), measured by flow cytometry, trans-well, and tube formation assay, respectively, after co-transfection with miR-33a-5p mimic and XBP1s-pcDNA3.1 at 48 h, followed by OGD treatment for 4 h. ***P<0.001 vs ov-NC.

Based on the previous results, we decided to investigate whether XBP1s may promote cell viability and angiogenesis in OGD-treated BMECs even under the interference of miR-33a-5p overexpression. Hence, miR-33a-5p mimic and XBP1s-pcDNA3.1 were co- transfected into BMECs and confirmed using qRT-PCR and western blot analysis. From the results, it can be seen that owing to the XBP1s-pcDNA3.1 transfection, XBP1s expression remained higher in the co-transfected miR-33a-5p mimic and XBP1s-pcDNA3.1 BMECs after the 4-h OGD treatment (Figure 10A). Conversely, DANCR and miR-33a-5p expression did not significantly change in the three groups (Figures 10B and 10C). Verification of our analysis revealed that cell viability, migration, and angiogenesis were significantly increased, whereas apoptosis was significantly reduced in the XBP1s+miR-33a-5p mimic group compared to those in the miR-33a-5p mimic or miR-33a-5p mimic+ov-NC groups owing to XBP1s overexpression (Figure 11).

### DANCR/miR-33a-5p/XBP1s activates the WNT/β-catenin signaling pathway in OGD-treated BMECs

**Figure 12:**
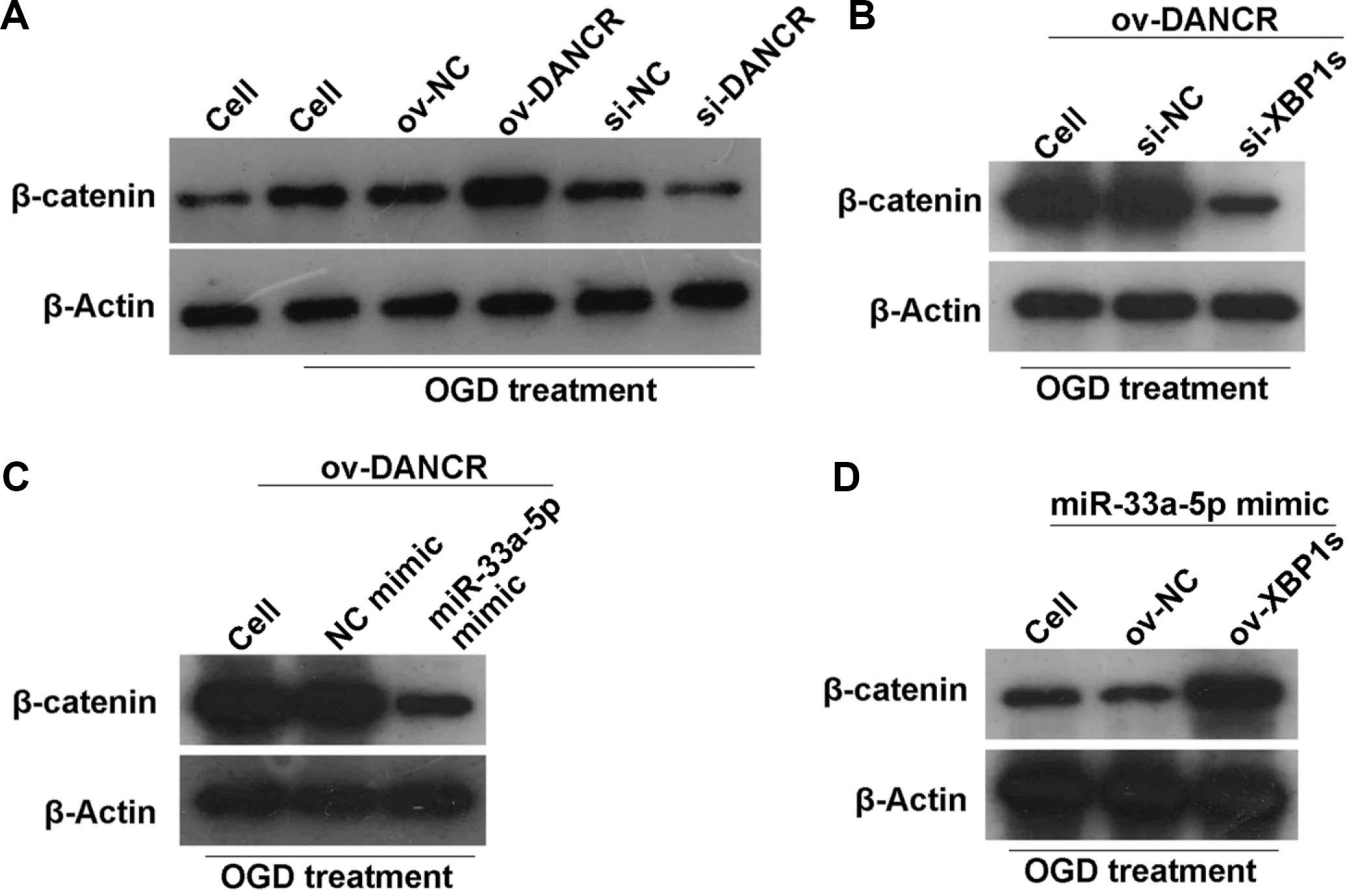
**DANCR/miR-33a-5p/XBP1s activates the WNT/β-catenin signaling pathway in OGD-treated BMECs.** (**A**) β-catenin expression was measured by western blotting after transfection of ov-NC, ov-DANCR, si-NC, and si-DANCR in OGD-treated BMECs. (**B**) β-catenin expression measured by western blotting after transfection of ov-DANCR+si-NC and ov-DANCR+si-XBP1s in the OGD-treated BMECs. (**C**) β-catenin expression measured by western blotting after transfection of ov-DANCR+NC mimic and ov-DANCR+miR-33a-5p mimic in OGD-treated BMECs. (**D**) β-catenin expression measured by western blotting after transfection of miR-33a-5p mimic+ov-NC and miR-33a-5p mimic+ov-XBP1s in the OGD-treated BMECs.

A previous study found that DANCR activates Wnt/β-catenin signaling to promote glioma proliferation [13]. In the present study, we found that β-catenin expression was significantly promoted by DANCR overexpression, whereas it was significantly inhibited by silencing DANCR expression in OGD-treated BMECs (Figure 12A). Additionally, β-catenin expression was significantly inhibited in the DANCR+si-XBP1s and DANCR+miR-33a-5p mimic groups compared with the DANCR+si-NC and DANCR+NC mimic groups, respectively (Figures 12B and 12C). Finally, we found that β-catenin expression was significantly promoted in the miR-33a-5p mimic+ov-XBP1s group compared with the miR-33a-5p mimic+ov-NC group (Figure 12D).

## DISCUSSION

Abnormal variants of lncRNAs are closely associated with stroke. In this study, we found that DANCR expression was upregulated in OGD-induced BMECs. Additionally, DANCR promoted BMEC proliferation and angiogenesis through the regulation of miR-33a-5p/XBP1s in OGD-induced BMECs, which may be important for ischemic stroke therapies.

Ischemic stroke induces BMEC death and BBB disruption and aggravates neurological injury [24]. BMEC angiogenesis is positively correlated with the survival rate of patients with stroke [25]. Therefore, promoting BMEC survival and angiogenesis after ischemic stroke may have great significance for improving the prognosis of stroke. In the present study, we found that DANCR promoted cell viability, cell migration, and angiogenesis, whereas silenced DANCR expression further inhibited cell viability, cell migration, and angiogenesis in OGD-treated BMECs. Additionally, DANCR overexpression enhanced XBP1s expression. In our previous study, we found that XBP1s, an important transcription factor, promotes BMEC survival and induces angiogenesis, thereby attenuating ischemia-induced BMEC injury [21]. In the present study, we found that knockdown of XBP1s expression reversed the effect of DANCR on proliferation and angiogenesis. These results strongly suggest that DANCR can attenuate OGD-induced cell damage by increasing XBP1s expression and that it plays a protective role in ischemic stroke.

We further investigated the mechanism of the DANCR effect. DANCR has been found to act as a ceRNA to sponge miRNAs. Previous studies have also identified that DNACR can sponge miR-33a-5p to regulate cell growth and metastasis in glioma and osteosarcoma [22, 23]. In line with the findings of these studies, we found that DANCR directly bound with miR-33a-5p in BMECs. miR-33a-5p overexpression inhibited proliferation and lumen formation by mediating oxidative stress, enhancing a homocysteine-induced cardiac microvascular endothelial cellular injury [26]. In colorectal cancer, miR-33a-5p suppressed cell growth, metastasis, and angiogenesis [27]. In the present study, we also demonstrated that miR-33a-5p overexpression inhibited proliferation, migration, lumen formation, and XBP1s expression and enhanced apoptosis in OGD-treated ov-DANCR-BMECs. These results demonstrate that miR-33a-5p reversed the protective effect of DANCR, synergistically enhancing BMEC apoptosis under ODG treatment. Taken together, our data demonstrate that DANCR promotes cell proliferation and angiogenesis through sponging miR-33a-5p in ODG-treated BMECs. Additionally, we found that the 3’-UTR of XBP1s directly bound with miR-33a-5p in BMECs. XBP1s attenuated OGD-induced BMEC injury in our previous study [21]. Herein, we also found that XBP1s overexpression promoted proliferation, migration, and lumen formation and recued apoptosis in OGD-treated miR-33a-5p-overexpressing BMECs. These results demonstrate that XBP1s reversed the damaging effect of miR-33a-5p and reduced BMEC apoptosis under ODG treatment. Taken together, our data demonstrate that miR-33a-5p inhibits cell proliferation and angiogenesis through the inhibition of XBP1s in ODG-treated BMECs.

Silencing of the Wnt/β-catenin pathway contributes to the development of ischemic stroke [28]. Activation of Wnt/β-catenin signaling contributes to the reduction of neuroinflammation, attenuation of BBB disruption, and facilitation of neurological recovery [29, 30]. A previous study found that DANCR activated Wnt/β-catenin signaling to promote glioma proliferation [13]. In the present study, we found that DANCR and XBP1s overexpression activated Wnt/β-catenin signaling, while miR-33a-5p overexpression silenced Wnt/β-catenin signaling. This suggests that Wnt/β-catenin signaling is a downstream signaling pathway regulated by DANCR/miR-33a-5p/XBP1s. DANCR/miR-33a-5p/XBP1s could activate Wnt/β-catenin signaling to attenuate BMEC damage induced by OGD.

There are several limitations to this study. First, we only explored the effect of miR-33a-5p on DANCR-regulated XBP1s expression. In fact, miR-33b-5p, miR-1251-5p, and miR-4731-5p may also play important roles. Additionally, miR-33a-5p may have more target genes other than XBP1s. Finally, the function of the DANCR/miR-33a-5p/XBP1s axis needs to be verified *in vivo*.

In conclusion, we found that DANCR enhanced the survival and angiogenesis in OGD-treated BMECs through the miR-33a-5p/XBP1s axis, which provides a better understanding of the roles of DANCR and miR-33a-5p in ischemic stroke. Our findings indicate that DANCR overexpression and miR-33a-5p knockdown exhibit a protective effect in ischemic stroke. Thus, DANCR and miR-33a-5p may be a therapeutic target for the treatment of stroke.

## MATERIALS AND METHODS

### Primary culture of rat BMECs and OGD treatment

This study was approved by the Animal Care Committee of Xiangya Hospital, Central South University, and all efforts were made to minimize the discomfort to the animals. Sprague-Dawley (SD) rats (aged 3–5 weeks) were fed as previously described [31]. Rat BMECs were isolated from SD rats according to the protocols detailed in our previous study [21]. Primary BMECs were cultured in Dulbecco’s modified Eagle medium with fetal bovine serum (FBS; 10%; GIBCO BRL, Gaithersburg, MD, USA) and incubated at 37°C, in humidified 5% CO_2_ and 95% air into a 75-cm^2^ flask. Three passages of BMECs were cultured in D-Hank’s medium, instead of normal culture medium, and incubated under hypoxic conditions (temperature 37°C, and atmosphere 95% N_2_ and 5% CO_2_). BMECs were exposed to OGD for 0, 2, 4, 6, 8, and 10 h.

### Quantitative real-time polymerase chain reaction assay

Quantitative real-time polymerase chain reaction (qRT-PCR) was used to evaluate the expression levels of DANCR, miR-33a-5p, and XBP1s. Total RNA was extracted from cultured cells using Trizol reagent (Invitrogen, Carlsbad, CA, USA). For gene expression analysis, 1 μg of total RNA was reverse transcribed in a final volume of 20 μL to synthesize first-strand cDNA using the ImProm-II reverse transcription system (Promega, Madison, WI, USA). qRT-PCR was performed using the SYBR® Premix ExTaq™ II kit (Takara, Dalian, China) on a 7500 Real-Time PCR System (Applied Biosystems; Thermo Fisher Scientific, Inc.). β-actin and U6 were used as endogenous controls to normalize mRNA/lncRNA and miRNA expressions, respectively. The relative expression levels of mRNA/lncRNA and miRNA were calculated using the 2^-ΔΔCq^ method. All reactions were performed in triplicate. The primer sequences used were as follows: DANCR-F: 5′-GAAAGTGCACCAAAGGGTAT-3′ DANCR-R: 5′-AGAATTGCCACCATTGTCTC-3′ XBP1s-F: 5′-AAGAAGAGAACCAGAAACTCC-3′ XBP1s-R: 5′-TGAGACCTCTTCAGTAACCA-3′ β-actin-F: 5′-AGGGAAATCGTGCGTGACAT-3′ β-actin-R: 5′-GAACCGCTCATTGCCGATAG-3′ miR-33a-5p-F: 5′-GTGCATTGTAGTTGCATTGCA-3′ miR-33a-5p-R: 5'-GTGCAGGGTCCGAGGT-3′ U6-F: 5′-GCTTCGGCAGCACATATACTAAAAT-3′ U6-R: 5′-CGCTTCACGAATTTGCGTGTCAT-3′.

### Western blot assay

Collected cells or tissues were homogenized in a radioimmunoprecipitation assay buffer. After, the homogenized solutions were centrifuged at 12 000 rpm for 15 min, and the supernatants were collected. Protein concentration was determined using the bicinchoninic acid assay. An equal amount of proteins was electrophoresed in 12% SDS-PAGE and then transferred to a polyvinylidene difluoride membrane and blocked with 5% non-fat milk in Tris-Buffered Saline Tween-20 (TBST) buffer for 1 h. Subsequently, the membranes were incubated with anti-XBP1s (ab220783, Abcam) antibodies at 1:500 dilution in 5% non-fat milk overnight at 4°C, followed by incubation with anti-rabbit antibodies conjugated with horseradish peroxidase (1/10000; Southern Biotech, Birmingham, AL, USA) at 1:2000 dilution for 1 h at 25°C, followed by three times washing with TBST. The protein bands were visualized on a gel-imaging system using ECL luminol reagent. β-actin was used as an internal reference to confirm the comparable amount of proteins in each lane.

### Cell transfection

miR-33a-5p mimic (5′-GUGCAUUGUAGUUGCA UUGCA-3′) and miRNA mimic negative control (NC mimic, 5′-TTCTCCGAACGTGTCACGT-3′) were purchased from GenePharma (Suzhou, China). DANCR sequence, which included Nhe1 and Xho1 restriction enzyme cutting sites, was chemically synthesized by GENEWIZ (Suzhou, China) and then cloned into pcDNA 3.1 (ov-DANCR). The empty pcDNA 3.1 plasmid served as a negative control (ov-NC). BMECs (2 ×10^5^ cells/well) were transfected with 1 μg/μL ov-DANCR without and with 50 nM miR-33a-5p mimic using 1 μL Lipofectamine® 2000 (Invitrogen; Thermo Fisher Scientific, Inc.), according to the manufacturer’s instructions. Additionally, three siRNAs for DANCR (i.e., si-DANCR-1, si-DANCR-2, and si-DANCR-3) and a negative control (si-NC) were synthesized by GenePharma, with the following sequences, si-NC: 5′-GAACUGGGGUGCGUGUGAUdTdT-3′; si-DANCR-1: 5′-CTGAATACTCTGCAGCTGCdTdT-3′; si-DANCR-2: 5′-GATTACACCCCCTTGTAAAdTdT-3′; si-DANCR-3: 5′-GGTGGAGAAGCCTGGCAGGdTdT-3'. Cells were transfected using lipofectamine RNAiMAX (Invitrogen). At 48 h after transfection, BMECs were exposed to OGD for 4 h.

### MTS assay, flow cytometry assay, migration assay, and tube formation assay

Cell proliferation was analyzed using the CellTiter 96 AQueous One Solution Cell Proliferation Assay kit (Promega), and Cell cycle detection kit (Keygen, Nanjing, China) and Annexin V-FITC apoptosis detection kit (Keygen) were used to detect the cell cycle and apoptosis rates, respectively. Migration assay and tube formation assay were analyzed according to the protocols detailed in our previous study (scale bar: 200×) [21].

### Statistical analysis

All experiments were performed in triplicate and data were expressed as mean ± standard deviation. Differences between the control and experimental groups were analyzed using the two-tailed Student’s t-test. An analysis of variance (ANOVA) was used for the overall comparison of the measurement indices between the groups, followed by post-hoc tests of the least significant difference (LSD). Differences of *p* < 0.05 (*); *p* < 0.01(**), or *p* < 0.001(***) were considered statistically significant.
